# Chitosan functionalisation of gold nanoparticles encourages particle uptake and induces cytotoxicity and pro-inflammatory conditions in phagocytic cells, as well as enhancing particle interactions with serum components

**DOI:** 10.1186/s12951-015-0146-9

**Published:** 2015-11-18

**Authors:** Matthew S. P. Boyles, Theresa Kristl, Ancuela Andosch, Mirjam Zimmermann, Ngoc Tran, Eudald Casals, Martin Himly, Victor Puntes, Christian G. Huber, Ursula Lütz-Meindl, Albert Duschl

**Affiliations:** Department of Molecular Biology, Division of Allergy and Immunology, Paris-Lodron University Salzburg, Hellbrunnerstr. 34, 5020 Salzburg, Austria; Department of Molecular Biology, Division of Chemistry and Bioanalytics, Paris-Lodron University Salzburg, Salzburg, Austria; Department of Cell Biology, Paris-Lodron University Salzburg, Salzburg, Austria; Institut Català de Nanotecnologia, Bellaterra, Barcelona, Spain; Institut Catala de Recerca i Estudis Avancats, Barcelona, Spain

**Keywords:** Charged gold nanoparticles, Chitosan, Exocytosis, Pro-inflammatory responses, Protein corona

## Abstract

**Background:**

Gold nanoparticles (AuNPs) are a popular choice for use in medical and biomedical research applications. With suitable functionalisation AuNPs can be applied in drug delivery systems, or can aid in disease diagnosis. One such functionalisation is with chitosan, which enables efficient interaction and permeation of cellular membranes, providing an effective adjuvant. As both AuNPs and chitosan have been shown to have low toxicity and high biocompatibility their proposed use in nanomedicine, either individually or combined, is expanding. However, further toxicological and immunological assessments of AuNP-chitosan conjugates are still needed. Therefore, we have evaluated how AuNP functionalisation with chitosan can affect uptake, cytotoxicity, and immunological responses within mononuclear cells, and influence the interaction of AuNPs with biomolecules within a complex biofluid. The AuNPs used were negatively charged through citrate-coating, or presented either low or high positive charge through chitosan-functionalisation. Uptake by THP-1 cells was assessed via transmission electron microscopy and electron energy loss spectroscopy, pro-inflammatory responses by ELISA and qRT-PCR, and cell death and viability via lactate dehydrogenase release and mitochondrial activity, respectively. Interactions of AuNPs with protein components of a frequently used in vitro cell culture medium supplement, foetal calf serum, were investigated using mass spectrometry.

**Results:**

Although cells internalised all AuNPs, uptake rates and specific routes of intracellular trafficking were dependent upon chitosan-functionalisation. Accordingly, an enhanced immune response was found to be chitosan-functionalisation-dependent, in the form of CCL2, IL-1β, TNF-α and IL-6 secretion, and expression of *IL*-*1β* and *NLRP3* mRNA. A corresponding increase in cytotoxicity was found in response to chitosan-coated AuNPs. Furthermore, chitosan-functionalisation was shown to induce an increase in unique proteins associating with these highly charged AuNPs.

**Conclusions:**

It can be concluded that functionalisation of AuNPs with the perceived non-toxic biocompatible molecule chitosan at a high density can elicit functionalisation-dependent intracellular trafficking mechanisms and provoke strong pro-inflammatory conditions, and that a high affinity of these NP-conjugates for biomolecules may be implicit in these cellular responses.

**Electronic supplementary material:**

The online version of this article (doi:10.1186/s12951-015-0146-9) contains supplementary material, which is available to authorized users.

## Background

Due to quantum size effects, and an increase in stability compared to other metal nanoparticles (NPs) [1], gold NPs (AuNPs) are one of the most promising materials utilised in nanotechnology. Amongst other areas, the extensive application of AuNPs in medicine and biomedical research is fast becoming a promising avenue of nanotechnology [2]. Aided by easy functionalisation [1] applications range from diagnostic imaging [3, 4] to the improved efficacy of drug and gene delivery systems [5–8], or the development of a novel “bedside tool” for cancer diagnosis [9] where functionalised AuNPs can recognise specific patterns of volatile organic compounds found in the breath of non-small-cell lung cancer patients [9, 10]. Other applications of AuNPs in nanomedicine involve functionalisation with chitosan, a molecule which itself has also been a focus of many medical applications [11]. Chitosan is a polysaccharide derivative of chitin, sourced from the seafood industry, which in recent years has been incorporated into medical research and practice due to low toxicity, high biocompatibility and its ability to interact and permeate cellular membranes, providing an effective adjuvant [12]. This topic is the focus of an extensive review series edited by Amidi and Hennink [13]. In respect to nanomedicine, chitosan has been proposed for use in gene therapy [14, 15], such as in siRNA [16], DNA [17, 18], and drug delivery systems [19, 20], and in cancer therapy [21, 22]. Conjugates of chitosan and AuNPs have been presented as a suitable tool for biosensing [23, 24], in drug delivery [25, 26], as antibacterial [27] and antifungal agents [28], and for tumour targeting [29]. Due to the relatively high level of deacetylation compared to chitin, chitosan is considered as hydrophilic [30]; in many applications it is this form which is used [23–27]. However, the development of nanomedicines utilising chitosan has also further processed this polymer to generate hydrophobic or amphiphilic particles [31]. While treatment efficacy is maintained [32], or even enhanced [33], blood circulation time may be extended with these novel materials [32], an enhanced cellular uptake efficiency [31, 34] and a wider intracellular distribution can be obtained, as well as the potential to avoid the unwanted degradation of the delivered package can be achieved through encouragement of alternative uptake mechanisms, such as macropinocytosis [31]. As AuNPs and chitosan are often considered inert and biocompatible, the proposed use of both agents in nanomedicine is expanding. For example, Au-chitosan NPs almost identical to those used in the present study are being recommended for enhanced insulin delivery via oral and nasal administration [26]. Furthermore, with an FDA classification of “generally regarded as safe” (GRAS), chitosan has been approved as a food additive and in wound dressing [35]. However, the resultant high localised concentrations possible during administration, warrants that these materials are considered more closely.

The available literature mostly corroborates the biocompatibility of AuNPs. For example, 3–8 nm lysine-capped AuNPs were shown to induce no detrimental effects in mouse macrophages in vitro [36]. Using air liquid interface (ALI) exposure, 15 nm citrate-coated AuNPs were found to induce no immune responses or anomalous redox activities in numerous cell types [37]. In both these studies AuNPs were internalised by cells. Furthermore, 3.7 nm pegylated AuNPs (therefore negatively charged) were even observed within the nucleus of HeLa cells with no apparent cytotoxicity [38]. However, other studies, discussed below, report conflicting results and the biocompatibility of AuNPs could not always be confirmed. It is at present not fully clear which aspects of Au-based NPs could be responsible for changing biologically inert NPs into potentially toxic NPs.

The current literature highlights various characteristics which may result in enhanced toxicity of AuNPs. Particularly small (<2 nm) Au-phenylphosphine NPs were found to be considerably more cytotoxic than larger (15 nm) AuNPs in numerous cell types [39]. An increase in cytotoxicity has been attributed to the presence of positive charges on the NP surfaces [40, 41]. In the study by Schaeublin et al. [40] both anionic and cationic NPs induced reactive oxygen species (ROS)-associated cytotoxicity. However, the increased cell death elicited by positively charged AuNPs was concomitant with a disruption of mitochondrial membrane potential and stimulation of intracellular calcium signalling [40]. Furthermore, chitosan-gold nanocomposites which have been developed for bactericidal applications were shown to considerably reduce the viability of mammalian cells [27]; this toxic effect was found to be dependent on numerous factors, and effects could be placated when using chitosan of differing molecular weight and degree of deacetylation, or by altering the original synthesis formulation, in terms of Au stock concentration [27]. A positive particle surface charge has often been linked to an increase in cellular uptake and concomitant increase in toxic potential of AuNPs [41]. Primary reticuloendothelial cells internalise greater quantities of positively charged AuNPs than negatively charged ones [42]. This is understandable as anionic particles display a limited or non-existent interaction with cell membranes, while an increase in cationic AuNPs’ toxicity can be mediated by an increase in interaction between cationic AuNPs and the negatively charged lipid bilayer [43]. To further complicate the assessment of biological responses to NP characteristics, the initial interaction of NPs with protein components of biofluids can alter cellular responses and NP characteristics [44–47].

In the present study we investigate the interaction of differently charged AuNPs with proteins in cell culture medium (CCM) and the biological consequences of this in mononuclear phagocytes, including uptake, cytotoxicity and inflammatory responses. We have therefore assessed (1) AuNP functionalisation-dependent internalisation by a human monocytic cell line (THP-1), (2) AuNP-induced cell death and inflammatory responses within the same cell line, and (3) the impact of chitosan-AuNP conjugates on interactions with foetal calf serum (FCS) components of standard in vitro CCM. Citrate-coated AuNPs of ~10 nm in diameter were used as synthesised or conjugated with different concentrations of chitosan, of particularly high deacetylation of 75–85 %, to give a range of surface charges (both negative and positive). The THP-1 cell line was chosen as it is a frequently used and versatile model both for monocytes and macrophages, which are two immune cell types with high phagocytic potential that are expected to take up and respond to NPs in many contexts.

## Results

### Nanoparticles

AuNPs were ~10 nm in diameter. These included one negatively charged sample (Au_SC, −45 mV ± 0.2), with the surface charge provided by the loose attachment of citrate ions. The remaining two samples (Au_CHIT-L and Au_CHIT-H) had increasing levels of positive charge (23 mV ± 1.0, 65 mV ± 1.0, respectively) provided by increasing concentrations of chitosan surface densities of 0.001, and 0.1 wt % (in solution). The intrinsic particle characteristics, upon synthesis, are provided in Table 1.

**Table 1:** Nanoparticle properties

Sample	Surface coating	Z-potential (mV)	Size nm	pH^a^	Concentration (mg/ml)	Solvent
Gold nanoparticles (AuNPs)						
Au_SC	SC^b^	−45 ± 0.2	10 ± 1.5	7	0.032	Sodium citrate 2.2 mM
Au_CHIT-L	0.001 % Chit.	+23 ± 1.0	7 ± 3	5	0.025	Chitosan 0.001 %^c^
Au_CHIT-H	0.1 % Chit.	+65 ± 1.0	7 ± 3	5	0.025	Chitosan 0.1 %

Sample ID, functionalisation, surface charge, size, pH in solvent, concentration of stock, and suspension solvent

^a^The acid or basic pH values turn to physiologically pH (around 7–8) when diluted at 1:10 (v/v) in cell culture medium

^b^ *SC* sodium citrate

^c^1 % = 1 g chitosan (chit.)/100 ml of solution

### Uptake and intracellular trafficking of AuNPs by THP-1 cells

**Fig. 1:**
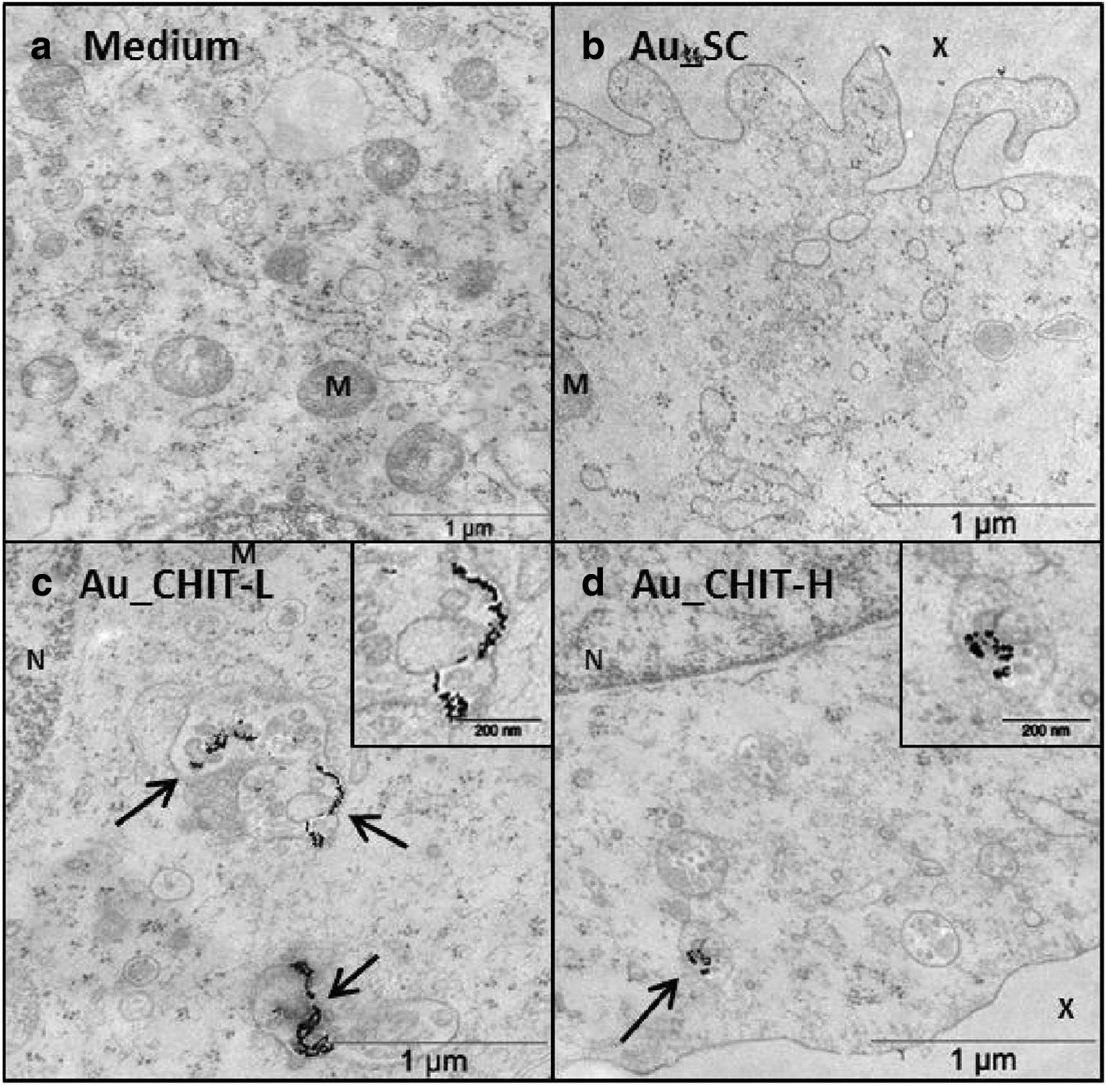
TEM micrographs of AuNP endocytosis by THP-1 cells. Images show 30 min incubation with **a** medium only, **b** Au_SC, **c** Au_CHIT-L, **d** Au_CHIT-H; AuNPs compartmentalised within endocytotic vesicles identified by *black arrows*; with nucleus, mitochondria and extracellular space identified by *N*, *M* and *X*, respectively

**Fig. 2:**
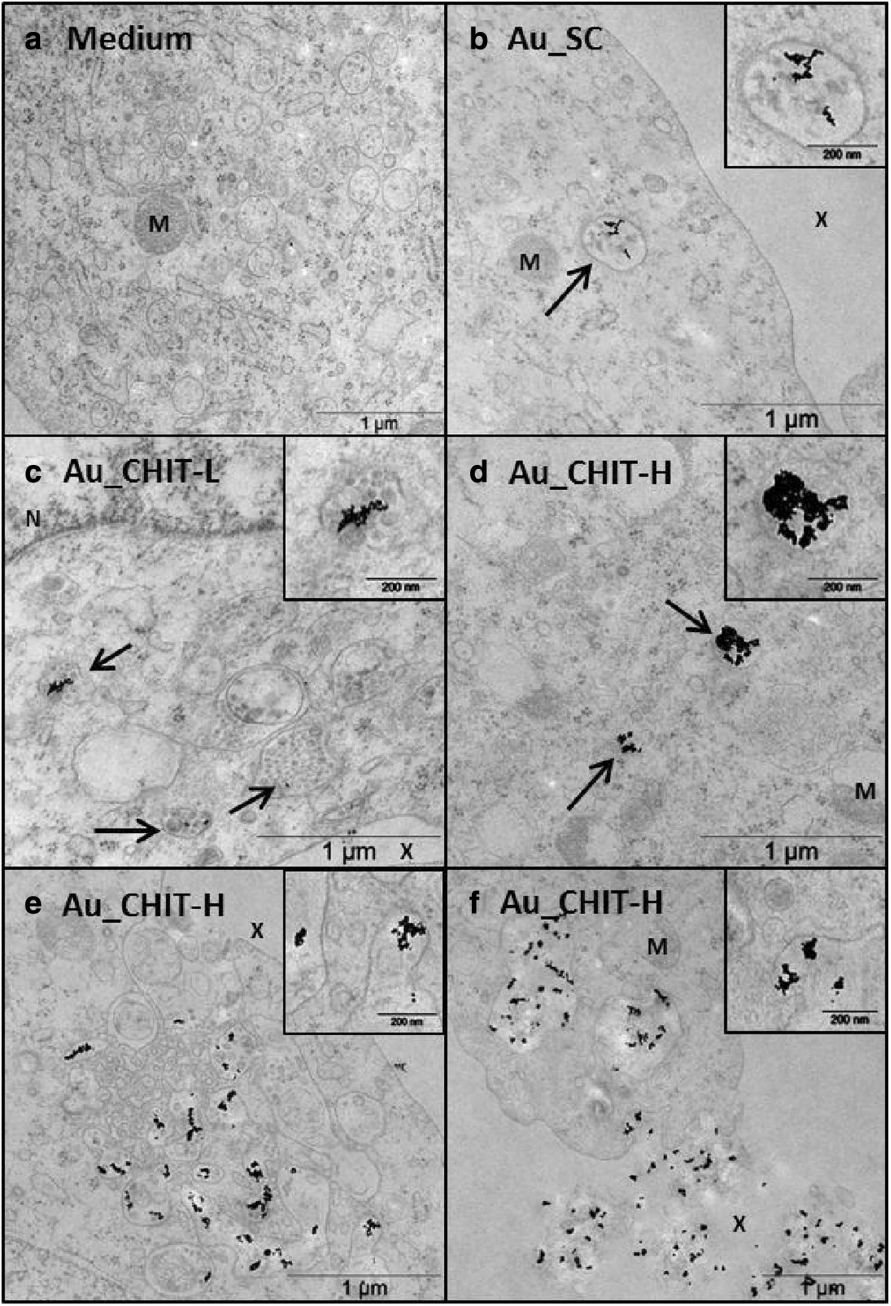
TEM micrographs of AuNP endocytosis and intracellular trafficking by THP-1 cells. Images show 6 h incubation with **a** medium only, **b** Au_SC, **c** Au_CHIT-L, **d** Au_CHIT-H; and Au_CHIT-H-containing vesicles shown fusing at the cell surface and eventual exocytosis (**e**, **f**); AuNPs compartmentalised within endocytotic vesicles identified by *black arrows*; with nucleus, mitochondria and extracellular space identified by *N*, *M* and *X*, respectively

The human monocytic cell line THP-1 was used to assess the uptake of functionalised AuNPs. Transmission electron microscopy (TEM) micrographs of 30 min and 6 h incubation of cells with AuNPs are shown in Figs. 1 and 2, respectively. After only 30 min both positively charged AuNPs were observed by TEM to be internalised. Small agglomerates could be seen within vesicles and not within the cytoplasm or other cell compartments. No observations of intracellular Au_SC could be made during this short exposure period. After 6 h exposure THP-1 cells were seen to have internalised all AuNPs types. All were observed within vesicles in different states of agglomeration, with Au_CHIT-H observed as larger agglomerates. In some cells, Au_CHIT-H were shown to be held within larger vesicles, and were also observed around the exterior of the cells. It can be seen in Fig. 2e, f that smaller NP-containing vesicles combined close to the cell surface, forming larger vesicles. As the extracellular NPs shown in Fig. 2f appeared to be accompanied with biological material, it was thought feasible that this fusion of lysosomes may ultimately have led to exocytosis of internalised AuNPs. At this time point, TEM micrograph examination of a cell which had released AuNP material revealed that the cell appeared to maintain a healthy condition (data not shown), indicating this to be an active process. The intracellular electron dense material observed in these TEM micrographs was confirmed to be Au by Energy Electron Loss Spectroscopy (EELS) (Additional file 1A–F), while similar electron dense material outside of the cell was not Au in all cases (Additional file 1E, F). The most likely explanation for these objects is that they are osmium, a contamination derived from the fixation process. However, electron dense material which was shown to be actively exocytosed by cells was confirmed as AuNPs (Additional file 1G, H). Within the electron energy loss (eV) spectra used for Au detection, there was also the appearance of phosphorus in most traces (Additional file 1B, D, F, H), which is to be expected due to the relatively high level of phosphorus within mammalian cells [48]; an observation corroborated by a disappearance of this peak in measurements taken extracellularly (Additional file 1E, F, green (control) trace).

### Increased cytotoxicity associated with chitosan-coating of AuNPs

**Fig. 3:**
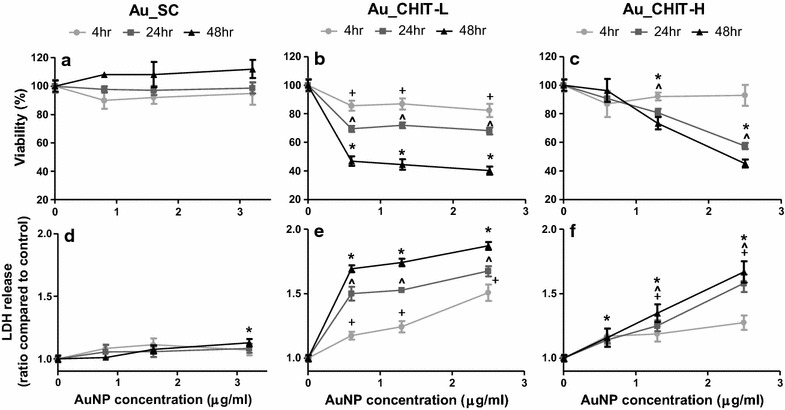
AuNPs induced cytotoxicity in THP-1 cells. Viability (mitochondrial activity) (**a**–**c**) and cytotoxicity (LDH release) (**d**–**f**) in PMA-stimulated cells, after treatment with Au_SC (**a**, **d**), Au_CHIT-L (**b**, **e**), and Au_CHIT-H (**c**, **f**), for 4, 24 and 48 h; 1 ng/ml LPS was used for co-stimulation, Triton X-100 was used as positive control. Results are expressed as, for viability,  % viability compared to 100 % control cells, and as the ratio change compared to controls for LDH release, and each data point represents the mean ± SEM of three independent biological replicates; statistical significance (determined by ANOVA with Tukey posthoc) is shown by +p < 0.05 for 4 h exposures, ^p < 0.05 for 24 h, and *p < 0.05 for 48 h, compared to relevant controls

THP-1 cells were primed with phorbol 12-myristate 13-acetate (PMA) prior to treatment with functionalised AuNPs. To determine cell death and viability, THP-1 cells were assessed for membrane integrity in the form of lactate dehydrogenase (LDH) release, and for mitochondrial activity using the CTB assay. Cells were exposed to Au_SC in a concentration range of 0.8–3.2 and 0.6–2.5 µg/ml for both Au_CHIT NPs, for 4 and 24 h (Fig. 3). Viability was unaffected by Au_SC, while a low but significant release of LDH was shown after 48 h exposure of the highest concentration of Au_SC. The cytotoxicity in response to Au_CHIT-L and Au_CHIT-H was more pronounced. A significant reduction in cell viability and release of LDH was both time- and dose-dependent in response to these two AuNPs. These responses were considerable and far greater than those to Au_SC. As there was an abrupt increase in cytotoxicity in response to Au_CHIT-L, lower concentrations were further assessed (Additional file 2). Solvent controls (sodium citrate and chitosan) at concentrations in accordance with particle exposures were also assessed for their influence of cell death and reduced viability, and were found to induce neither (Additional file 3).

### Inflammation and inflammasome activation correlate with chitosan-coating of AuNPs

THP-1 cells were used to assess the induction of a pro-inflammatory environment. Initially, the response of THP-1 cells to AuNPs under different cell activation states was investigated using Au_CHIT-H only. Cells were treated with Au_CHIT-H for 4 and 24 h in the presence or absence of lipopolysaccharide (LPS), and with or without PMA pre-stimulation. The resulting supernatant was analysed for secretion of IL-1β. Without PMA pre-stimulation (Additional file 4A, B) IL-1β was significantly induced at both time points when LPS co-stimulation was used. Without LPS co-stimulation IL-1β secretion was found only at the longer exposure period of 24 h. The 4 h exposure of Au_CHIT-H in the absence of LPS co-stimulation induced a 2.6-fold increase in IL-1β secretion compared to control cells, however, this was not found to be statistically significant (p = 0.072). When PMA pre-stimulation was used (Additional file 4C, D), IL-1β secretion was observed at both time points when LPS was included, but was found only after 4 h when LPS was absent. At 24 h, in the absence of LPS, Au_CHIT-H did induce a 4.5-fold increase in IL-1β secretion compared to medium only control cells. Again, this was not found to be significant (p = 0.086). Each experimental protocol used here demonstrated the pro-inflammatory response which developed upon exposure of AuNPs coated with chitosan. Due to the increased sensitivity for IL-1β secretion of PMA-primed cells with additional LPS co-stimulation, it was decided to use this system to further study Au_CHIT NP-induced inflammation.

**Fig. 4:**
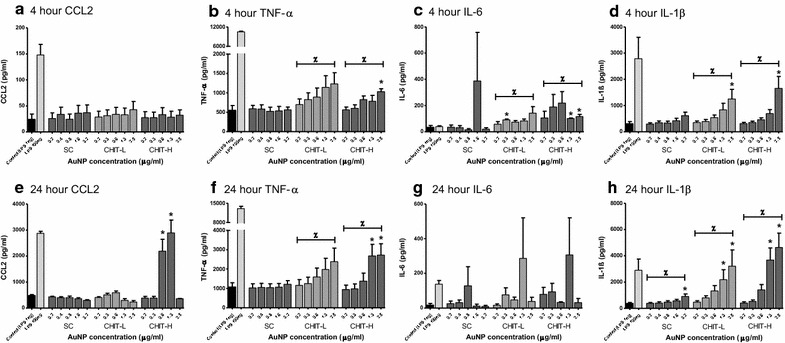
AuNPs induced pro-inflammatory response of THP-1 cells. Secretion of CCL2 (**a**, **e**), TNF-α (**b**, **f**), IL-6 (**c**, **g**) and IL-1ß (**d**, **h**) from PMA-stimulated cells, in response to Au_SC, Au_CHIT-L, and Au_CHIT-H; 1 ng/ml LPS was used for co-stimulation; with 1 and 100 ng/ml LPS for controls; results are expressed as cytokine release in pg/ml, and each data point represents the mean ± SEM of three independent biological replicates; statistical significance (determined by ANOVA with Tukey posthoc) is shown by *p < 0.05, compared to relevant controls (1 ng/ml LPS); or by Spearman’s rank correlation coefficient to assess the NP dose dependent relationship with cytokine release, where Ζ = p < 0.05

PMA-stimulated THP-1 cells were exposed to all functionalised AuNPs in a concentration range of 0.2–3.2 µg/ml for Au_SC and 0.2–2.5 µg/ml for both Au_CHIT, for 4 and 24 h, with LPS co-stimulation of 1 ng/ml. The resulting supernatants were assessed for CCL2, IL-1β, TNF-α and IL-6. No significant release of CCL2 was found after 4 h particle exposures (Fig. 4a), while after 24 h (Fig. 4e) a significant CCL2 secretion was shown with treatments of 1.25 and 2.5 µg/ml Au_CHIT-H. This effect was shown to be even higher than for the 100 ng/ml LPS positive control. None of the other AuNPs used induced CCL2 secretion. Release of TNF-α was found to dose-dependently increase in response to both Au_CHIT NPs during 4 (Fig. 4b) and 24 h (Fig. 4f), an effect not seen in Au_SC exposures. A similar dose dependency in treatments of Au_CHIT NPs was observed in secretion of IL-6 after 4 h (Fig. 4c), which was no longer evident after 24 h exposure (Fig. 4g). Au_SC did not stimulate significant IL-6 release. A dose-dependent increase in IL-1β was observed with exposure of both chitosan-coated AuNPs after 4 h (Fig. 4d), and to all AuNPs after 24 h (Fig. 4h). During these exposure periods Au_CHIT-L and Au_CHIT-H were shown to be particularly proficient in inducing IL-1β secretion. Inflammatory responses of THP-1 cells were also assessed in response to relevant concentrations of the AuNP solvents (sodium citrate and chitosan solutions) and no significant responses were observed (Additional file 5).

With respect to the high IL-1β secretion, further experiments were performed to assess what impact differently charged AuNPs have on specific stages of the IL-1β secretory pathway. These included the expression of *IL*-*1β* and *NLRP3* mRNA, and determination of inflammasome activation. PMA-primed THP-1 cells were exposed to Au_SC at 3.2 µg/ml and both Au_CHIT at 2.5 µg/ml in the presence and absence of 1 ng/ml LPS (for co-stimulation). For gene expression 1 and 24 h time points were used, while 4 and 24 h were used for determination of inflammasome activation. For determination of NLRP3 inflammasome involvement these exposures were also performed in the presence and absence of the caspase-1 inhibitor Ac-YVAD-CMK.

**Fig. 5:**
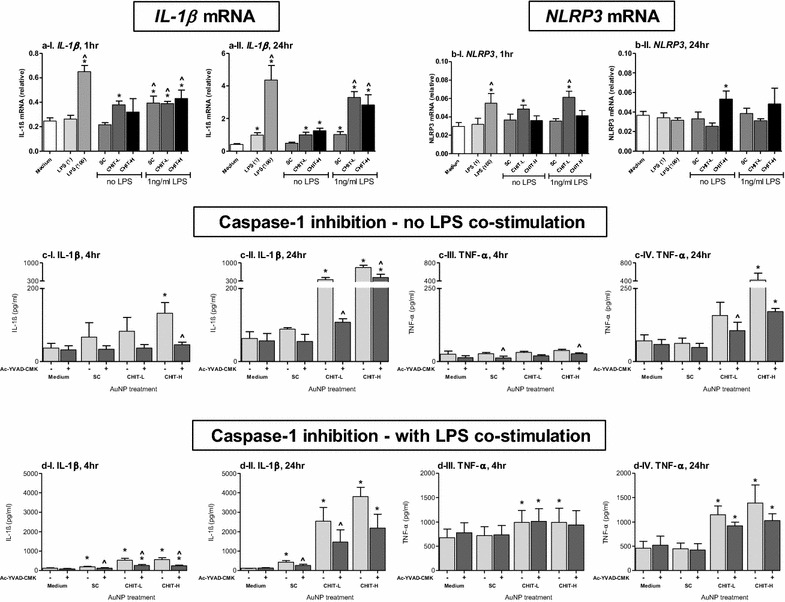
NLRP3 inflammasome involvement in AuNP pro-inflammatory responses. THP-1 cells were treated with AuNPs at 3.2 (Au_SC) or 2.5 (Au_CHIT) µg/ml. mRNA of (**a**) IL-1β and (**b**) NLRP3 was determined by qRT-PCR after 1 and 24 h exposure; results are expressed in relation to the RPLP0 housekeeping gene, and each data point represents the mean ± SEM of at least three independent biological replicates; statistical significance (determined by ANOVA with Tukey posthoc) of particle treatments compared to controls is shown by *p < 0.05 for medium only, and ^p < 0.05 for 1 ng/ml LPS. The effect of Ac-YVAD-CMK (caspase-1 inhibitor) on AuNP-induced IL-1β and TNF-α secretion was determined by ELISA after 4 and 24 h; AuNP exposures were performed in **c** absence or **d** presence of LPS co-stimulation; results are expressed as cytokine release (pg/ml), and each data point represents the mean ± SEM of at least three independent biological replicates. Statistical significance (determined by ANOVA with Tukey posthoc) is shown by *p < 0.05 compared to relevant negative controls, and (determined by T-Test) with ^p < 0.05 when comparing corresponding inhibited and uninhibited exposures

In the absence of LPS co-stimulation *IL*-*1β* mRNA was found to be elevated in response to Au_CHIT-L and Au_CHIT-H at both times measured (Fig. 5a). All *IL*-*1β* mRNA levels were shown to be significantly greater than controls (medium only treated cells) except the 1 h exposure of Au_CHIT-H. With the inclusion of LPS co-stimulation, *IL*-*1β* gene expression was significantly elevated (compared to LPS-treated control cells) in response to all AuNPs after 1 h, and only to Au_CHIT NPs after 24 h. The *IL*-*1β* gene expression observed after 24 h in response to Au_CHIT-L and Au_CHIT-H, with and without LPS co-stimulation, was notably higher than with Au_SC treatments. *NLRP3* mRNA (Fig. 5b) was significantly increased only in response to Au_CHIT-L during the 1 h incubation period, and only towards Au_CHIT-H during the 24 h exposure period.

In the absence of LPS co-stimulation the level of secreted IL-1β protein increased in response to Au_CHIT-H after 4 h (Fig. 5c-I) and to both Au_CHIT NPs after 24 h (Fig. 5c-II). These responses were significantly reduced when cells were pre-treated with Ac-YVAD-CMK. In the case of 24 h Au_CHIT-H exposure, IL-1β was still significantly elevated when compared to relevant control (Ac-YVAD-CMK-treated cells). Under the same conditions, Ac-YVAD-CMK pre-treatment induced significantly lower levels of TNF-α upon stimulation with Au_SC and Au_CHIT-H for 4 h, albeit within these exposures TNF-α secretion was not found to be significantly higher than for control cells in response to any NPs (Fig. 5c-III). After 24 h Au_CHIT-H induced significant TNF-α secretion, regardless of Ac-YVAD-CMK inclusion (Fig. 5c-VI). With the inclusion of 1 ng/ml LPS co-stimulation, all AuNPs induced significant IL-1β release at both time points tested (Fig. 5d-I and -II). IL-1β release was found to be particularly prominent in response to both Au_CHIT-L and Au_CHIT-H at each time point. Ac-YVAD-CMK pre-treatment was shown to reduce IL-1β secretion in response to all AuNPs and at both time points. TNF-α was significantly increased in response to both Au_CHIT NPs after 4 h, with no Ac-YVAD-CMK-dependent effects observed (Fig. 5d-III). With 24 h exposure the high response elicited by Au_CHIT-L and Au_CHIT-H was maintained, and although Ac-YVAD-CMK was shown to lower these responses, no significant decrease was observed (Fig. 5d-IV). Au_SC induced no TNF-α secretion.

### Chitosan surface density influences AuNP interactions with cell culture medium proteins

**Fig. 6:**
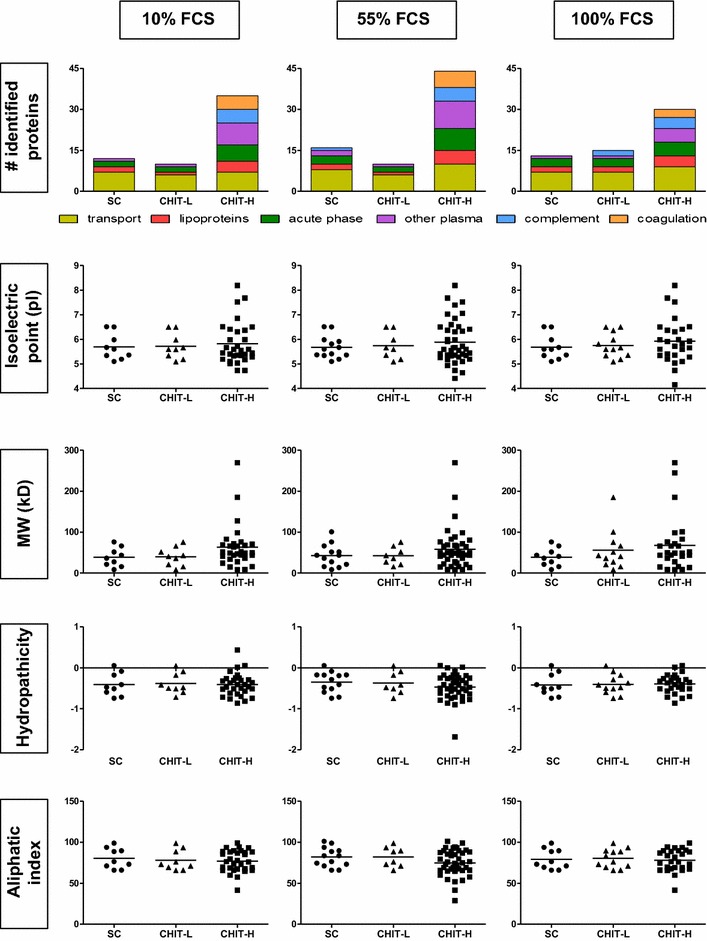
Characteristics of FCS proteins associating with AuNPs. The number of unique proteins identified, via LTQ-Orbitrap mass spectrometry, in AuNP-FCS complexes were characterised by their functional groupings, isoelectric point, molecular weight, hydropathicity and aliphatic index; evaluated when incubated in (*left column*) 10 %, (*centre column*) 55 %, and (*right column*) 100 % FCS; data presented is of unique proteins identified in all 3 biological replicates; protein functional classification is based on http://www.Uniprot.org, protein parameters were determined using the ProtParam tool of http://www.expasy.org; this analysis was performed with three technical replicates of each biological replicate; for confidence of identification the proteins presented in these *graphs* are only proteins found in every biological replicate

AuNPs were incubated in the presence of 10, 55 or 100 % FCS for 24 h and subsequently washed and isolated via centrifugation. The number and identity of proteins associated with NPs can be viewed in Additional files 6, 7 and 8. Further classification of proteins was according to their biological function, isoelectric point (pI), molecular weight (MW), hydropathicity (GRAVY) and aliphatic index (Fig. 6). The proteins presented in Fig. 6, and in Additional files 6, 7 and 8 are the unique proteins which appeared in every biological replicate, as this was considered a good quality control, and infers that these are the proteins which would routinely interact with these AuNPs. However, in addition to this, the unique proteins which were found associated with AuNP-protein complexes not only in all biological replicates, but also in all FCS concentrations, are presented in Additional files 9 and 10.

An increase in the number of unique proteins associating with AuNPs was found to be mainly dependent on AuNP functionalisation with a particularly high concentration of chitosan (Fig. 6). There was little difference observed between Au_SC and Au_CHIT-L. However, when the concentration of chitosan on the NP surface increased in Au_CHIT-H, so did the number of unique proteins. Au_SC were found to accumulate slightly more proteins than Au_CHIT-L, except when NPs were incubated in 100 % FCS, in which case a strict charge dependency was observed. Protein functional categories were assigned as: lipoproteins, proteins associated with transport, acute phase responses, complement, coagulation, or other plasma proteins. Using this classification the increase in proteins associating with Au_CHIT-H was determined to be an increase in proteins of all functional groups. Particularly striking were increases in other plasma proteins, and proteins associated with complement, coagulation and acute phase responses. These additional FCS proteins were not only shown to be functionally different, they were also found to be characteristically different. Out of the four protein parameters evaluated (pI, MW, GRAVY and aliphatic index), the hydropathicity of proteins associating with AuNPs remained similar. However, a greater range of protein pI, MW and, to a smaller extent, aliphatic index was observed in the proteins which associated with AuNPs of particularly high chitosan density.

## Discussion

The focus of this study was to determine how AuNP functionalisation can (I) influence uptake and (II) inflammatory responses in mononuclear phagocytic cells, and (III) alter the interaction of proteins with these NP conjugates. Negatively charged citrate-stabilised AuNPs were compared to AuNPs containing different surface densities of positively charged chitosan.

### AuNP endocytosis and intracellular trafficking by THP-1 cells is dependent on AuNP functionalisation

Internalisation of NPs has been shown to be dependent on size [36, 49–51], aggregation state [52], surface charge (and/or functionalisation) [41, 53, 54], or simply on cell phenotype [41, 50, 52]. Here we have shown that rapid internalisation of AuNPs by THP-1 cells was driven by chitosan coating of AuNPs. While positively charged AuNPs already internalised within 30 min, negatively charged AuNPs were only evident inside cells after 6 h. This is in line with much of the literature concerning charged NPs, as cationic AuNPs have been shown to keenly associate with negatively charged lipid bilayers [43], to be internalised rapidly [55], and to enter cells via alternative mechanisms compared to anionic NPs [56]. Rapid endocytosis of cationic AuNPs has been reported to occur within 30 min [57], and even as early as 5 min [58], while in both these cases endocytosis of anionic AuNPs was not evident at the respective time points. It should be noted that it is possible that these differences were due to the poor colloidal stability, and therefore aggregation and rapid sedimentation of cationic NPs [59]. However, a more likely explanation is the increased affinity of cationic NPs for the negatively charged cell membrane [57], an occurrence also postulated by Hühn et al. [41] in the exposure of fibroblasts with both negatively and positively charged AuNPs.

Besides uptake rates, AuNP surface charge has been shown to influence intracellular trafficking. We have shown that with eventual endocytosis of all NP charge states, NP agglomerates were held within endocytic vesicles. Similar observations have been made elsewhere, including internalisation of lysine-capped AuNPs by macrophages [36]. However, in the study by Shukla et al. [36], intracellular trafficking resulted in a perinuclear arrangement of AuNP agglomerates. Furthermore, Ojea-Jiménez et al. [57] also observed endosomes containing cationic AuNPs migrating towards the nucleus, and additionally, rupturing of the endosomes in proximity of nuclei, leaving AuNPs available to enter the nucleus. The explanation given for this behaviour was the proton sponge hypothesis [57], a mechanism which was also shown to occur in monocyte-derived macrophages exposed to cationic amino-functionalised PSNPs (45.1 mV), and not in response to anionic PSNPs [60]. It is considered that cationic NPs present within a lysosome undergoing acidification may accumulate protons entering via the proton pump, therefore acting as a “proton sponge” and maintaining the pump active, resulting in osmotic swelling, lysis and release of the lysosome content into the cytoplasm [44]. We did not observe AuNPs free within the cytoplasm, and therefore it is unlikely that our cationic AuNPs induced a “proton sponge” effect, neither did they accumulate near the cells’ nucleus. Instead the vesicles containing highly positively charged Au_CHIT-H, and this type of NP alone, were found to accumulate near and fuse with the outer cell membrane. The fusion of lysosomes with the plasma membrane and subsequent exocytosis of ingested material is a recognised pathway [61], and involves membrane repair during the procedure [62], therefore it may not necessarily elicit further cell stress or death, but is often reported in conjunction with intracellular calcium signalling [62, 63], a signalling event known to be involved in NP-induced pro-inflammatory responses [64–66]. With this in mind, and as we observed cells undergoing this process to be in good health, it seems feasible that the extracellular AuNPs identified, and associated with biological material, may have been exocytosed by cells. If so, our observations imply that this specific mechanism of exocytosis was dependent on NP surface charge, a conclusion which was also made by Oh et al. [67], who observed a similar response of cells to positively charged AuNPs. NPs would exit the cell in large agglomerates combined with intracellular material. A noteworthy point is that while Oh et al. [67] demonstrated exocytosis within 48 h using positively charged AuNPs with a zeta potential closer to Au_CHIT-L, we have shown a potential route for exocytosis within a far shorter exposure time to AuNPs with a far greater zeta potential. This implies that the level of NP charge which is presented may play an important role in exocytosis. As many intracellular applications of NPs such as drug delivery systems rely upon vesicle transport routes, the implications of this observed exocytosis are important to consider, as the intrinsic toxicity of a material for the whole organism may increase. These expulsions would include lysosome material, which itself could trigger unwanted biological effects, and NP material which could lead to further cell interactions and secondary endocytosis. It should be noted that although we, and Oh et al. [67], propose NP charge as a controlling factor for AuNP exocytosis, other studies have also implicated NP size in determining exocytosis [51].

### Inflammation and cytotoxicity driven by AuNP functionalisation

Macrophages are among the principal immune effector cells, therefore responses of this cell type are considered paramount in determining biological responses to medically relevant NPs. Here we have demonstrated that within our phagocytic models, AuNP functionalisation with chitosan plays a crucial role in mediating AuNP-induced cell death and cellular inflammatory responses, which were well aligned with the uptake patterns previously discussed. The initial phagocyte model used in this study was that of the THP-1 monocytic cell line, in which we identified a rapid uptake of AuNPs dependent upon the functionalisation with chitosan. As one goal of this study was to assess inflammasome activation in response to functionalised AuNPs it was thought prudent to assess IL-1β secretion within different activation states of our phagocyte model, as the differential regulation of this protein is considered to differ between monocytes and macrophages [68]. This was restricted to a combination of PMA-priming and LPS co-stimulation, as this is a method commonly used in studies involving NLRP3 inflammasome activation in THP-1 cells [68–70]. We observed the highest release and greatest sensitivity for IL-1β secretion when cells were both PMA-primed and co-stimulated with LPS, and therefore this condition was used in subsequent experiments. As we did not further assess the internalisation of AuNPs after these activation steps were included, we can only postulate as to how uptake would have been affected. Subtle differences in NP uptake have been reported within phagocytes, with primary macrophages internalising greater quantities of anionic polystyrene NPs (PSNPs) than THP-1 cells; while the reverse was true for cationic PSNPs, as THP-1 cells were shown to endocytose greater quantities than primary macrophages. These differences were driven by the use of different mechanisms of uptake; phagocytosis was employed by macrophages, while uptake by undifferentiated THP-1 cells was driven by dynamin II-dependent endocytosis, or by macropinocytosis in PMA-differentiated THP-1 cells [53]. This may imply that the differences in pro-inflammatory cytokine release in response to different cell activation procedures may be influenced by uptake mechanisms in place of actual quantity of internalised AuNPs. However, using AuNPs of particularly high positive surface charge Bartneck et al. [71] observed uptake within macrophages to be considerably greater than in monocytes. It is, therefore, feasible that in our case the enhanced activation state of the PMA-primed THP-1 cells resulted in a higher pro-inflammatory mediator release due to distinct mechanisms employed resulting in an enhanced level of AuNP uptake.

A change of negative to positive charge on silica NPs (SiNPs) corresponded with an increase in cell death and oxidative stress in macrophages [72]; an outcome also observed by Hühn et al. [41] in the treatment of HUVECs and C17.2 cells with charged AuNPs. In contrast, upon exposure of human keratinocytes, AuNPs were shown to induce cell death irrespective of whether the NPs held a positive, negative or neutral charge [40]. However, the extent of cell death was greater when NPs were charged, as was the mechanism of cytotoxicity [40]. It must be noted that the study by Schaeublin et al. [40] used concentrations of NPs far greater than those used here. Our results are in line with these studies, as we have shown that a switch from negative to positive surface charge can induce significantly greater cell death in THP-1 cells. Moreover, using Au-chitosan composites formed of chitosan of a similar molecular weight to the one used in our study, Regiel-Futyra et al. [27] have previously shown a reduction of cell viability in epithelial cells (A549) comparable to that found here in phagocytic cells (THP-1). However, the concentrations used by Regiel-Futyra et al. were far higher than those used here and their NP size was larger than those used in the present study. Regiel-Futyra et al. found no toxic effects when the NP concentration was lowered. This provides an indication of cell-specific sensitivities and/or NP size-dependent responses. Notably, Regiel-Futyra et al. were able to mollify these responses through using chitosan of different molecular weights. As their nanocomposites were found to possess considerable bactericidal activity, these results highlight an important step in modern development of nanotherapeutics, where subtle changes in formulation can dramatically ameliorate the effect upon mammalian cells while maintaining their functional purpose. The responses of THP-1 cells we observed at far lower NP concentrations compared to those identified in A549 cells by Regiel-Futyra et al. could be explained by cell-specificity in terms of NP uptake, which raises the question of appropriate cell type choice when assessing medically relevant NMs. Hühn et al. [41] demonstrated that while uptake of negatively charged AuNPs was unchanged across different cell phenotypes, the internalisation of positively charged ones was significantly different dependent on cell type; an observation also found elsewhere [50, 52, 73]. This can be attributed to the use of different uptake mechanisms employed [50], even when considering monocytes against macrophages [53]. In terms of NP uptake in THP-1 versus A549, it is difficult to elucidate any conformity within the literature. THP-1 cells have been shown to accumulate a greater quantity of SiO_2_NPs than A549 cells, and at a faster rate [74]. However, the NP uptake which was quantified by Mohamed et al. [74] was based on anionic NPs. A549 cells have been shown to readily internalise chitosan-coated AuNPs [75], and as non-phagocytic cells are considered to have a preference for cationic NPs compared to anionic ones [76], uptake of chitosan coated NPs by A549 could be potentially greater. Moreover, epithelial cells have been shown to internalise greater quantities of cationic NPs than macrophages [77]. In this study by Xia et al. [77], uptake induced significant cell death in both cell types. However, the mechanisms were found to be different and dependent on surface charge. With apoptosis induced in macrophages through lysosomal rupture, and necrosis induced in epithelial cells upon internalisation through caveolae-dependent mechanisms [77]. Furthermore, in a study by Hsiao et al. [78] comparative NP uptake rates between A549 and THP-1 cells alternate dependent on NP size and exposure dose. It is therefore difficult categorically assign why we have observed this difference in sensitivity between our THP-1 responses and those of the A549 cells used by Regiel-Futyra et al. however, we place the most likely explanation upon mechanisms of internalisation.

In a study by Arvizo et al. [58] the proliferation and viability of human bronchial epithelial cells and human airway smooth muscle cells dosed with positively charged 10 nm AuNPs was found to be considerably hampered through the induction of apoptosis, with corresponding fluctuations in intracellular calcium signalling. These events were not found in exposure of neutral or negatively charged AuNPs [58]. Changes in intracellular calcium levels such as this would imply that positively charged AuNPs have the potential to stimulate greater inflammatory responses than negatively charged NPs. Here, we have shown that the secretion of the pro-inflammatory cytokine IL-1β was considerable in response to Au_CHIT NPs, which also induced high levels of *IL*-*1β* gene expression. These data led us to examine the involvement of the NALP3 inflammasome in charged AuNP-induced inflammatory responses. This mechanism is stipulated by many studies concerning particle-induced inflammatory responses, including investigations into carbon nanotubes, amorphous silica, nano-TiO_2_ and asbestos [79–82]. The direct involvement of Au_CHIT-L and Au_CHIT-H in NALP3 inflammasome activation was confirmed here through the significant reduction of AuNP-induced IL-1β secretion with the caspase-1 inhibitor Ac-YVAD-CMK, and the enhanced expression of *NLRP3* mRNA. Other pro-inflammatory markers assessed were CCL2, TNF-α and IL-6. TNF-α and IL-6 were secreted in response to both Au_CHIT NPs, while CCL2 only in response to Au_CHIT-H. None of these pro-inflammatory markers were induced by Au_SC. These data suggest that cellular pro-inflammatory responses were driven by NP surface charge, a finding corroborated elsewhere. IL-1β secretion by monocyte-derived macrophages was shown by Lunov et al. [60] to be dependent on a positive surface charge, and pegylated Au nanorods (PEG-AuNRs) with a RGD peptide motif addition were shown to induce an increase in TNF-α and MIG in primary macrophages when compared to PEG-AuNRs lacking this motif [42]. However, in the described studies, the amino-functionalised PSNPs were not able to induce TNF-α [60], the RGD motif was not able to induce IL-6 or IL-1β, and no charge dependency was shown for CCL2 secretion [42]. We observed a charge-dependent upregulation of all these factors, which would suggest that our observed pro-inflammatory responses were not only driven by charge, but also specifically by chitosan. This speculation was further strengthened by considering responses observed by Shukla et al. [36], who found neither TNF-α nor IL-1β in response to positively charged lysine-capped AuNPs in mouse macrophages. Shukla et al. [36] used AuNPs of a similar size to ours, and in higher concentrations. However, it is difficult to attribute these pro-inflammatory responses to the addition of chitosan alone as not only did control experiments show a lack of pro-inflammatory responses towards exposure of our NP solvents, but chitosan oligomers at a concentration comparable to that found in these NP exposures actually reduce pro-inflammatory responses [83]. It is more likely that a factor contributing to the high cytokine release shown here is the rapid endocytosis of Au_CHIT-L and Au_CHIT-H, and the perceived exocytosis of Au_CHIT-H. It is feasible that there was secondary internalisation of the exocytosed Au_CHIT-H, an event which has previously been demonstrated for 30 nm negatively charged peptide-functionalised AuNPs [84].

### Effect of AuNP functionalisation on biomolecule interactions

Interactions between NPs and biofluid components occur immediately and ultimately result in a stable corona surrounding the NP [47, 85, 86].This corona has been identified, in place of the particle itself, to play a major role in cellular responses, including cell attachment, inflammation, ROS generation, and cell death [45, 47, 85–87]. In fact, an interaction of cationic NPs with serum proteins has been shown to enhance cell binding, while reduced cell interactions were shown when NPs were anionic, inducing different mechanisms of uptake [56]. Therefore, it was considered that the difference in cellular response to the different functionalised AuNPs used in this study may, in part, be governed by the interactions occurring between these AuNPs and components of the CCM. We have focussed on protein components of FCS, as this is a supplement of CCM in the majority of in vitro studies. Furthermore, it allowed a comparative study of AuNP charge/functionalisation-related interactions with proteins within a complex mixture, which represents a central consideration as the formation of the protein corona determines the fate of NPs and strongly depends on the initial NP surface characteristics.

Although negatively charged NPs experience electrostatic repulsion with the majority of serum proteins, we found that Au_SC were still able to attract numerous proteins. This is reported to be likely due to the highly electrolytic media screening of these negative charges through the efficient decoration of both NP and protein surfaces with Ca^2+^ ions [88], allowing their close interaction, and subsequent reorientation and reorganization encouraging permanent interactions [89]. Our highly positively charged AuNPs were shown to accumulate a far greater number of proteins as their surface charge increased. Again this is understandable as cationic NPs attract proteins rapidly and with poor selectivity, thus leading to an increasing number of unique proteins, hindering effective reorganization and impeding corona hardening [89]. Nevertheless, due to most serum proteins being anionic, a stronger and faster association between the cationic AuNPs when compared to the anionic can be expected [57]. All AuNP charge states used here were shown to accumulate a common subset of FCS proteins including serum albumin, serotransferrin, alpha-1-acid glycoprotein, alpha-1-antiproteinase, alpha-1B-glycoprotein, alpha-2-HS-glycoprotein, hemoglobin fetal subunit beta, vitamin D-binding protein, apolipoprotein A-I, apolipoprotein A-II and fetuin-B. However, as the AuNP charge was altered from negative to increasingly positive, an increased number of unique proteins associated with the AuNP-protein complexes was identified. More specifically, these additional unique proteins were identified as proteins associated with complement, coagulation and acute phase responses, as well as other plasma proteins. Furthermore, these proteins were also shown, in part, to differ in pI and MW compared to the common subset. The data presented here on protein attachment to AuNPs highlight the number of different/unique proteins associating with NPs; other studies have quantified total protein amounts. Using a well-defined adsorption study into the attachment of serum albumin to the surface of negatively and positively AuNPs, Hühn et al. [41] have shown that a corona formed of this protein is unaffected by surface charge, in terms of protein number and affinity. However, this phenomenon may not apply to all NP coatings, as citrate-coated NPs have been shown to generate particularly strong binding with serum albumin [90]. Hence, it is possible that this is the reason for the lower abundance of proteins, other than albumin, found in our Au_SC-associated protein complexes. Alternatively, the lower binding efficiency of the citrate-coated NPs used here may be due to an intrinsic charge dependency of AuNPs within a complex protein mixture. While Hühn et al. [41] investigated interactions of AuNPs with albumin alone, it was shown by Deng et al. [86] that within a complex protein solution an increase in bound protein was concomitant with a switch from negatively to positively charged AuNPs. Further similarities between the data presented here and those of Deng et al. [86] were apparent. With an increasing density of positively charged functional group, chitosan in our study and poly[N-(2-aminoethyl)acrylamide] for Deng et al. [86], an increase in the number of bound proteins was observed. Furthermore, Deng et al. [86], as did we, found that a far lower number of unique proteins would associate with citrate-coated AuNPs compared to NPs with other coatings. Tenzer et al. [91] have shown that negatively charged SiNPs predominately bind negatively charged blood plasma proteins. Here the negatively charged Au_SC were also shown to exclusively bind negatively charged proteins (with pI < 7). Protein-NP complexes formed of highly positively charged Au_CHIT-H, although predominately associating with proteins of pI < 7, were also found in conjunction with proteins of pI > 7, indicating the attachment of positively charged proteins. This signifies that protein pI is in some way associated with attachment to highly charged AuNP-protein complexes, while other characteristics are less important than this macromolecular electrostatic interplay.

Within AuNP-protein complexes formed of Au_CHIT-H we identified various molecules associated with cellular interaction and internalisation, including hyaluronan-binding proteins such as anti-thrombin III, inter-α-trypsin inhibitor heavy chains, and complement, which have already been implicated as mediators of AuNP-cell interactions [87]; fibrinogen, which has been implicated in specific NP-cell interactions and subsequent immune responses [86, 92]; and transferrin, which has often been associated with NP internalisation, with transferrin-dependent clathrin-mediated endocytosis being a method employed within drug delivery systems [93]. This mechanism has previously been highlighted with exposure of various mammalian cells to AuNPs [51], and with the intrinsic exocytotic recycling of the transferrin receptor [93] may provide an interesting avenue for AuNP intracellular trafficking mechanisms. However, transferrin was found bound to all AuNPs used here, although quantities have not been determined and may vary between different preparations. In summary it is evident that the highly positively charged NPs used in the present study interact with many proteins within FCS which may contribute to the observed biological responses and unique intracellular trafficking.

## Conclusions

We have shown that differently functionalised AuNPs evolve both extracellularly and inside the cell, which consequently affects their biological impact. Here, the effect of the NP surface charge and charge intensity, through chitosan functionalisation, has been observed to radically affect the interactions with proteins, the intracellular fate of AuNPs, and consequently the cellular responses. We have consistently shown that the interaction with, and the toxicity induced in, cells of the mononuclear phagocyte system was driven by AuNP functionalisation with chitosan, and that increasing chitosan density can exacerbate these events; with enhanced uptake, enhanced cellular responses, and furthermore, a potential rapid and unique exocytosis. Moreover, we have demonstrated that AuNP functionalisation with chitosan, a molecule perceived as non-toxic and biocompatible, encourages a high affinity of AuNPs for biomolecules, which in turn led to cellular toxicity induced by two seemingly non-toxic NP-conjugate components. Therefore, it would be unwise to still consider all AuNPs, of any size, shape or surface composition, as completely inert drug carriers. Final formulations as a whole have to be assessed, since high density functionalisation with otherwise inert components may elicit potentially detrimental biological responses.

## Methods

### Materials

RPMI 1640, l-glutamine, streptomycin, penicillin, glutaraldehyde, lipopolysaccharide (LPS), Phorbol 12-myristate 13-acetate (PMA), Tris(2-Carboxyethyl)phosphine hydrochloride solution (TCEP), iodoacetamide (IAA), Bradford reagent, BSA, acetonitrile (ACN) trifluoroacetic acid (≥99.5 %, TFA), and chitosan (with >75 % deacetylation, #417963), were purchased from Sigma-Aldrich (Sigma, St. Louis, MI, USA). With the following materials purchased elsewhere: HEPES and FCS (PAA, Pasching, Austria), SYBR Green Supermix (Bio-Rad), RevertAid H Minus M-MulV reverse transcriptase (MBI Fermentas, St. Leon-Roth, Germany), TRIzol reagent (Invitrogen), CCL2, TNF-α and IL-6 ELISA kits (PeproTech), IL-1-β ELISA (R&D Systems), caspase-1 inhibitor Ac-YVAD-CMK (Calbiochem), epon (Agar Scientific), osmium tetroxide (Electron Microscopy Sciences, Hatfield, UK), triethylammonium bicarbonate buffer (TEAB) (Fluka, Buchs, Switzerland). While trypsin, the LDH detection kit CytoTox 96^®^ Non-Radioactive Cytotoxicity Assay, and the CellTiterBlue^®^ (CTB) Cell Viability Assay were purchased from Promega (Madison, WI, USA).

### Gold nanoparticle synthesis and characterisation

Negatively and positively charged AuNPs were synthesised with adaptions of methods described by Turkevich et al. [94] and Jana et al. [95], respectively. Synthesis and characterisation have previously been described [89, 96], and included size determination using TEM (JEOL 1010 electron microscope, Japan) and surface charge by zeta potential measurements using a Malvern ZetaSizer Nano ZS (Malvern Instruments, Malvern, UK).

### THP-1 cell culture

Cells were purchased from the European Collection of Cell Cultures (ECACC), and maintained at 2–8 × 10^5^ cells/ml under sterile conditions at 37 °C and 5 % CO_2_, using RPMI 1640 general culture medium, supplemented with 2 mM l-glutamine, 100 μg/ml streptomycin, 100 IU/ml penicillin, 10 mM HEPES, and 10 % FCS. Cells were maintained for no longer than 20 passages, and were seeded for particle exposures at a density of 1.6 × 10^5^ cells/cm^2^, to remain within the supplier recommended conditions. THP-1 cells were used either in their normal monocyte phenotype, or primed using PMA to allow a morphology and behaviour closer to that of macrophage-like cells. This transition was monitored by observation only, with cells becoming adherent and spread, opposed to the rounded, suspended monocytes. Co-stimulation with LPS was used to mimic the response of macrophages already undergoing inflammatory responses.

### Uptake and trafficking

For TEM, THP-1 cells were treated with an administered concentration of 3.2 µg/ml for Au_SC and 2.5 µg/ml for Au_CHIT for either 30 min or 6 h. To ensure the exposure medium remained at physiological pH the AuNP stock solutions needed a ten-fold dilution in CCM. Therefore, the highest concentrations used here (and those of the following experiments) were based on this necessity. Cells were then fixed with 1 % glutaraldehyde (50 mM) and subsequently washed using 0.05 M cacodylate buffer (pH7.2). Post-fixation was performed with 2 % osmium tetroxide (OsO_4_) in cacodylate buffer overnight at 4 °C. After washing with cold water, sample dehydration was performed at 4 °C with sequential increases of ethanol concentrations, starting at 10 %. When an ethanol concentration of 70 % was reached incubation with 1 % uranyl acetate in 70 % ethanol was included before continuation of ethanol dehydration up until 100 % ethanol. After dehydration, cells were embedded using the following solutions: a 1:1 (by volume) ethanol:propylene oxide mix, followed by propylene oxide alone, and finally a 1:1 (by volume) propylene oxide:epon (a low viscosity epoxy resin) mix. TEM micrographs were obtained of ultrathin sections (approx. 70 nm) of embedded cell suspensions. Cell structural assessment and determination of AuNP uptake and intracellular trafficking were investigated in a LEO 912 AB Omega transmission electron microscope (Zeiss, Oberkochen) operated at 80 kV with a LaB6 cathode. Micrographs were taken in conventional TEM mode. EELS of AuNPs was performed with ultrathin sections of embedded cell suspensions (approx. 40 nm) and analysed at 120 kV, using a magnification of 31,500 times, a spectrum magnification of 125 times, an illumination angle of 3.15 mrad, and exposure time of 50 s. Spectra and TEM micrographs were captured using a dual speed CCD Slow Scan Camera TRS Sharpeye (Troendle, Moorenwies, Germany) and processed in iTEM Olympus Software (Münster, Germany).

### THP-1 processing for determination of inflammatory responses

Different cell activation states were initially investigated for pro-inflammatory mediator release induced by 2.5 µg/ml Au_CHIT-H. Cells were pre-stimulated with 500 nM PMA 24 h prior to experiments, or left un-stimulated. This was followed by treatment of Au_CHIT-H for 4 and 24 h in the presence or absence of 1 ng/ml *Escherichiacoli*-derived LPS. Treatments of medium only and LPS at 1 ng/ml served as negative controls, while or 100 ng/ml LPS was used as a positive control. Supernatants were assayed for IL-1β by ELISA. PMA priming and LPS co-stimulation were chosen to investigate further pro-inflammatory mediators using all AuNPs described in this study. THP-1 cells were treated with Au_SC at 0.2–3.2 µg/ml and Au_CHIT at 0.2–2.5 µg/ml for 4 and 24 h. Supernatants were assayed for CCL2, IL-1β, TNF-α and IL-6 by ELISA. The same controls were used as described above.

The impact of AuNPs on the complex mechanism of IL-1β secretion was further investigated. One h prior to AuNP exposure, PMA pre-stimulated THP-1 cells were incubated with 100 μM of the caspase-1 inhibitor Ac-YVAD-CMK for 1 h in serum-free medium, to inhibit the activation of the NLRP3 inflammasome. Then in medium containing FCS, cells were treated with all AuNPs (Au_SC at 3.2 µg/ml and Au_CHIT at 2.5 µg/ml) for 4 and 24 h in the presence or absence of 1 ng/ml LPS. Supernatants assayed for TNF-α and IL-1β by ELISA. The same controls were used as described above.

### Determination of cyto-/chemokine release

ELISA was performed for each protein with an adaption of the manufacturer’s instructions. Briefly, the capture antibody, reconstituted in PBS, was added to wells of a 96 well plate at 0.25 (CCL2), 1 (TNF-α and IL-6) or 4 (IL-1-β) µg/ml, and left at 4 °C overnight. Subsequently, plates were kept at room temperature. Wells were then washed 3 times with wash buffer (0.05 % Tween20 in PBS) before addition of blocking buffer (1 % BSA in PBS) for 1 h. Following another wash step, standards, at 0-1000 (CCL2), 0-2000 (TNF-α and IL-6) or 0-250 (IL-1-β) pg/ml, and samples were added for 2 h, with subsequent wash step. Detection antibody, diluted to 500 (CCL2, TNF-α and IL-6) or 200 (IL-1-β) ng/ml in assay diluent (0.05 % Tween-20 and 0.1 % BSA in PBS), was added for 2 h, and plates were subsequently washed. An avidin-HRP conjugate was added for 30 min. A final wash step was performed, followed by the addition of a TMB substrate. After the reaction was stopped, using 2 M H_2_SO_4_, the plates were measured on a plate reader (Infinity 200 Pro, Tecan, Groedig, Austria) at 450 nm with a reference wavelength of 650 nm, and protein concentrations determined with use of protein standard curves.

### Determination of inflammasome activation

For determination of *IL*-*1β* and *NLRP3* gene expression induced by AuNPs total RNA was isolated from cells using TRIzol reagent and cDNA was generated with RevertAid H Minus M-MulV reverse transcriptase, following the manufacturer’s instructions. Quantitative real-time RT-PCR (qRT-PCR) was performed using a Rotorgene 3000 (Corbett Research, Mortlake, Australia), with iQ SYBR Green Supermix and the following primers: human IL-1β sense, 5′-GTACCTGAGCTCGCCAGTGA-3′,and antisense, 5′-TCGGAGATTCGTAGCTGGATG-3′; human NLRP3 sense, 5′-TCAGCACTAATCAGAATCTCACGCACCTTT-3′, and antisense, 5′-CCAGGTCATTGTTGCCCAGGCTC-3′; and human RPLP0 sense, 5′-GGCACCATTGAAATCCTGAGTGATGTG-3′, and antisense, 5′-TTGCGGACACCCTCCAGGAAG-3′. The large ribosomal protein P0 (RPLP0) was used as a reference gene, and PCR specificity was confirmed through assessment of the PCR product melting curves. Quantification of relative mRNA expression levels were calculated in relation to the RPLP0 housekeeping gene using the delta delta method of Pfaffl [97].

### Cytotoxicity and cell viability

After AuNP exposures, supernatants were collected for determination of released LDH. Fresh medium was added to wells and cell viability was then determined using the CellTiterBlue^®^ (CTB) Cell Viability Assay. CTB reagent was added at a ratio of 1:5 and cells were place at 37 °C for 60 min, after which the fluorescence intensity was measured at ex560/em590 on a plate reader (Infinity 200 Pro, Tecan, Groedig, Austria). LDH was determined in a colorimetric reaction using the CytoTox 96^®^ Non-Radioactive Cytotoxicity Assay. After collection, the supernatants were centrifuged for 10 min at 25000*g*, 30 µl was transferred to transparent 96-well plates, followed by 30 µl assay substrate. Plates were then incubated at room temperature for 20 min prior to addition of 30 µl stop solution, followed by absorbance measurements at 490 nm using a plate reader.

### Protein corona analysis

For protein binding experiments, three concentrations were used; 10 % FCS, as used in standard in vitro CCM, 55 % FCS, as this is the plasma percentage within whole blood, and 100 % FCS as a control for full protein content. AuNPs were incubated in the presence of FCS for 24 h and subsequently washed three times and isolated via centrifugation. The number and identity of proteins present, associated with NPs or NP agglomerates, were characterised via LTQ-Orbitrap mass spectrometry (MS). Each sample was dissolved in 50 µl 0.5 mol/l TEAB, followed by denaturation at 60 °C for 30 min. Protein concentrations were then determined by the Bradford assay. Disulfide bonds were reduced by adding 4.5 µl of a 50 mM TCEP-HCl, followed by 1 h incubation at 60 °C. The samples were alkylated by adding 8.7 mM IAA for 30 min at RT, protected from light. Tryptic digestion was performed overnight at 37 °C, using trypsin at a ratio of 1:50 in relation to protein concentration. The following day samples were purified with C18 Tips (according to the manufacturer’s instructions, Thermo Fisher Scientific, Bremen, Germany) to remove NPs and salts. After which, each sample was adjusted to a concentration of 1 µg/µl through drying and dissolving in mobile phase A (ultrapure water with 0.050 % TFA). The sample constituents were separated by nano-ion-pair reversed-phase—HPLC (U3000 nano, Dionex, Germany) at pH 2 and detected by LTQ-Orbitrap-MS (LTQ Orbitrap XL, Thermo Fisher Scientific, equipped with a nano-electrospray ionization source). The flow rate was set to 1 µl/min and a poly-styrene/divinylbenzene (PS-DVB) monolithic 150 × 0.2 mm I.D. column (produced in-house according to Premstaller et al. [98]) was used for separation. A 2 h gradient of 0–40 % ACN in 0.050 % TFA at 55 °C was applied. To identify peptides, three data-dependent collision-induced dissociation (CID) scans were performed. The MS1 survey scans of the eluting peptides were executed in the Orbitrap with a resolution of 60,000, recording a window between m/z 450 and 2000. To identify peptides, three data-dependent collision-induced dissociation (CID) scans of the precursor ions were carried out in the ion trap. The normalised collision energy (NCE) was set at 35 % for all CID scans. The FCS samples were measured three times with the use of exclusion lists. This procedure facilitated the identification of more proteins compared to using a single measurement. The data were analysed with Proteome Discoverer™ (Thermo Fisher Scientific, Version 1.3). Following parameters for the Spectrum Selector node were set: min. precursor mass 350 Da; max. precursor mass 5000 Da; s/n threshold 1.5. Parameters for Mascot (in-house server: version 2.3.2.) searches were as follows: precursor mass tolerance 10 ppm; fragment mass tolerance 0.5 Da; Trypsin; 1 missed cleavage site; Uniprot (taxonomy: mammalia) knowledgebase; dynamic modifications: oxidation (M) and deamidation (NQ); fixed modification: carbamidomethylation (C). The Peptide Validator tool was used for the processing node and the target false discovery rate (FRD) value was set to 0.01 (strict) and 0.05 (relaxed). Proteins were further characterised by their biological function, isoelectric point (pI), molecular weight (MW), hydropathicity (GRAVY) and aliphatic index, using the ProtParam tool and the UniProt knowledgebase of http://www.expasy.org. The data collected for the AuNP interactions with FCS proteins were obtained with three independent biological replicates and three technical replicates of each biological replicate.

### Statistical analysis

Statistical analysis of data was performed using PASW statistics 18 (SPSS, IBM), and treatments were considered statistically significant when p < 0.05. Differences between exposures of AuNPs and those of relevant controls (medium only, or 1 ng/ml LPS) were determined by ANOVA, with post hoc Tukey comparisons for pairwise analysis. Specific comparisons (i.e. treatments with and without caspase-1 inhibition) were made using an Independent Samples *T* Test. Relationships between AuNP dose and cytokine secretion were further assessed using the Spearman rank-order correlation coefficient.

## Additional files

10.1186/s12951-015-0146-9 EELS measured at sites of electron dense particulate matter observed in micrographs. Intracellular particles are identified with red lines (A–F), extracellular particles with green lines (E–F), and particles observed during perceived exocytosis with red lines (G–H); Au = gold, Co = control, P = phosphorus.
10.1186/s12951-015-0146-9 Au_CHIT-L induced cytotoxicity in THP-1 cells. Viability (mitochondrial activity) (A) and cytotoxicity (LDH release) (B) in PMA-stimulated cells, after treatment with Au_CHIT-L, for 24 and 48 h; 1 ng/ml LPS was used for co-stimulation, Triton X-100 was used as positive control. Results are expressed as, for viability,  % viability compared to 100 % control cells, and as the ratio change compared to controls for LDH release, and each data point represents the mean ± SEM, R = 4; statistical significance (determined by ANOVA with Tukey posthoc) is shown by ^ = p < 0.05 for 24 h, and * = p < 0.05 for 48 h, compared to relevant controls.
10.1186/s12951-015-0146-9 Cytotoxicity assessment of NP solvents in THP-1 cells. Viability (mitochondrial activity) (A-C) and cytotoxicity (LDH release) (D-F) in PMA-stimulated cells, after treatment with sodium citrate (2.2 mM stock) or chitosan (0.1 % stock) at dilutions relevant to AuNP exposures, for 4, 24 and 48 h; 1 ng/ml LPS was used for co-stimulation, Triton X-100 was used as positive control. Results are expressed as fluorescence intensity for viability assays, and as absorbance for LDH release, R = 3, each data point represents the mean ± SEM.
10.1186/s12951-015-0146-9 AuNPs induced pro-inflammatory response of THP-1 cells in different cell activation states. IL-1β release from THP-1 cells in response to treatment with Au_CHIT-H (administered dose of 2.5 µg/ml) for 4 (A and C) or 24 h (B and D), in the presence and absence of 1 ng/ml LPS co-stimulation, and without (A-B) or with (C-D) PMA-priming. Results are expressed as IL-1β release (pg/ml), and each data point represents the mean ± SEM, R = 3. Statistical significance is shown by * = p < 0.05, ** = p < 0.01 and *** = p < 0.005, compared to relevant controls (medium only or 1 ng/ml LPS); 100 ng/ml LPS was used as positive control.
10.1186/s12951-015-0146-9 Immune response of THP-1 cells to NP solvents. Secretion of CCL2 (A-C), TNF-α (D-F), IL-6 (G-I) and IL-1ß (J-L) from PMA-stimulated cells, in response to sodium citrate (2.2 mM stock) or chitosan (0.1 % stock) at dilutions relevant to AuNP exposures; 1 ng/ml LPS was used for co-stimulation; with 1 and 100 ng/ml LPS for controls; results are expressed as cytokine release in pg/ml, R = 3, and each data point represents the mean ± SEM.
10.1186/s12951-015-0146-9 Proteins bound to AuNP when using 10 % FCS. The identity of unique proteins identified, via LTQ-Orbitrap mass spectrometry, in AuNP-FCS complexes were identified using http://www.uniprot.org (taxonomy: mammalia); evaluated when incubated in 10 % FCS; data presented is of unique proteins identified in all 3 biological replicates; this analysis was performed with 3 technical replicates of each biological replicate; for confidence of identification the proteins presented in these graphs are only proteins found in every biological replicate.
10.1186/s12951-015-0146-9 Proteins bound to AuNP when using 55 % FCS. The identity of unique proteins identified, via LTQ-Orbitrap mass spectrometry, in AuNP-FCS complexes were identified using http://www.uniprot.org (taxonomy: mammalia); evaluated when incubated in 55 % FCS; data presented is of unique proteins identified in all 3 biological replicates; this analysis was performed with 3 technical replicates of each biological replicate; for confidence of identification the proteins presented in these graphs are only proteins found in every biological replicate.
10.1186/s12951-015-0146-9 Proteins bound to AuNP when using 100 % FCS. The identity of unique proteins identified, via LTQ-Orbitrap mass spectrometry, in AuNP-FCS complexes were identified using http://www.uniprot.org (taxonomy: mammalia); evaluated when incubated in 100 % FCS; data presented is of unique proteins identified in all 3 biological replicates; this analysis was performed with 3 technical replicates of each biological replicate; for confidence of identification the proteins presented in these graphs are only proteins found in every biological replicate.
10.1186/s12951-015-0146-9 General pattern of AuNP-protein interactions – number of unique proteins. The number of unique proteins identified, via LTQ-Orbitrap mass spectrometry, in AuNP-FCS complexes were identified using http://www.uniprot.orf (taxonomy: mammalia); evaluated when incubated in 10, 55, and 100 % FCS; data presented is of unique proteins identified in every biological replicate and all 3 serum conditions.
10.1186/s12951-015-0146-9 General pattern of AuNP-protein interactions – identity of unique proteins. The identity of unique proteins identified, via LTQ-Orbitrap mass spectrometry, in AuNP-FCS complexes were identified using http://www.uniprot.org (taxonomy: mammalia); evaluated when incubated in 10, 55, and 100 % FCS; data presented is of unique proteins identified in every biological replicate and all 3 serum conditions.

## Abbreviations

ALI: air liquid interface
Au_CHIT-L: chitosan coated gold nanoparticles-low density chitosan coating
Au_CHIT-H: chitosan coated gold nanoparticles-high density chitosan coating
AuNPs: gold nanoparticles
Au_SC: citrate coated gold nanoparticles
CCM: cell culture medium
CID: collision-induced decay
EELS: energy electron loss spectroscopy
FCS: foetal calf serum
GRAS: generally regarded as safe
GRAVY: grand average of hydropathicity
HPLC: high-performance liquid chromatography
LDH: lactate dehydrogenase
LPS: lipopolysaccharide
MS: mass spectrometry
MW: molecular weight
NCE: normalised collision energy
PMA: phorbol 12-myristate-13-acetate
ROS: reactive oxygen species
TEM: transmission electron microscopy
