# Supplementary figures and images for: Chitosan functionalisation of gold nanoparticles encourages particle uptake and induces cytotoxicity and pro-inflammatory conditions in phagocytic cells, as well as enhancing particle interactions with serum components
Source: J Nanobiotechnology. 2015 Nov 18;13:84. doi: 10.1186/s12951-015-0146-9 (PMC4652435; doi:10.1186/s12951-015-0146-9)

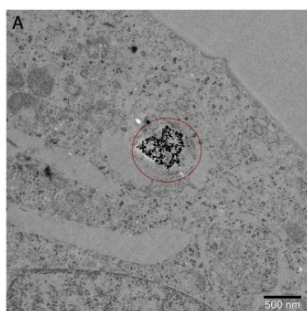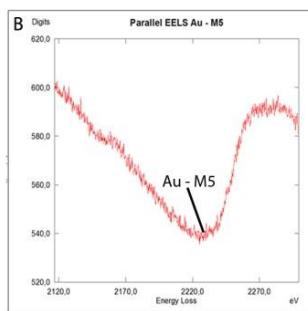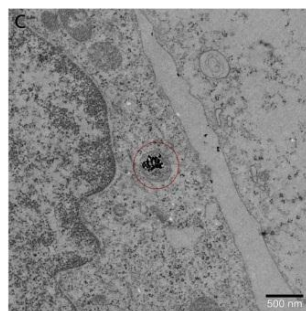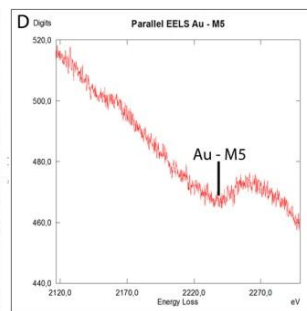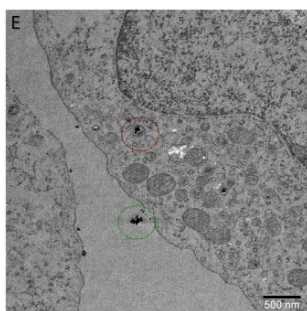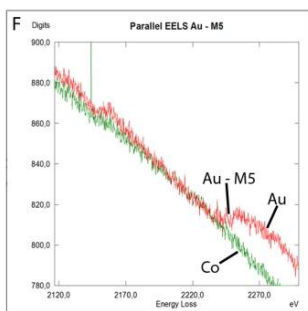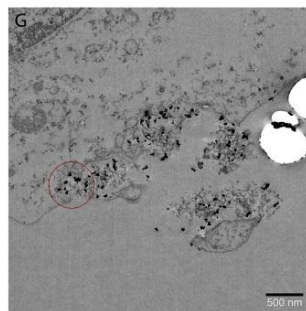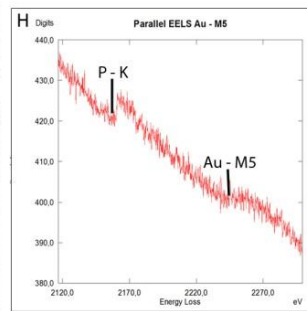

Supplement: Supplementary file 1 — 10.1186/s12951-015-0146-9 EELS measured at sites of electron dense particulate matter observed in micrographs. Intracellular particles are identified with red lines (A–F), extracellular particles with green lines (E–F), and particles observed during perceived exocytosis with red lines (G–H); Au = gold, Co = control, P = phosphorus. [file 12951_2015_146_MOESM1_ESM.pdf]

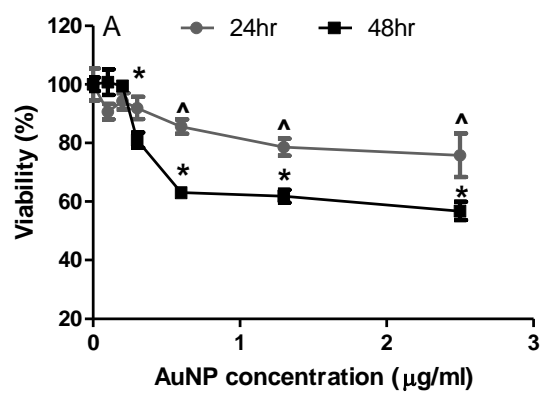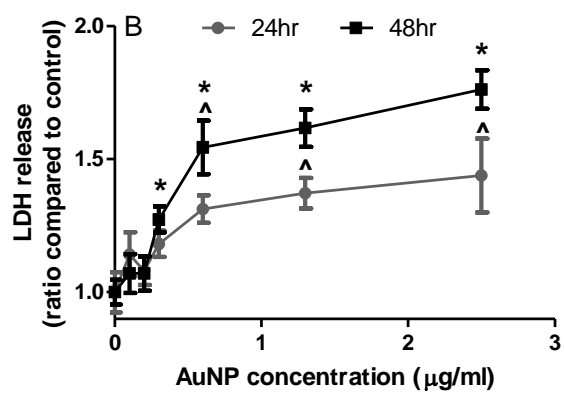

Supplement: Supplementary file 2 — 10.1186/s12951-015-0146-9 Au_CHIT-L induced cytotoxicity in THP-1 cells. Viability (mitochondrial activity) (A) and cytotoxicity (LDH release) (B) in PMA-stimulated cells, after treatment with Au_CHIT-L, for 24 and 48 h; 1 ng/ml LPS was used for co-stimulation, Triton X-100 was used as positive control. Results are expressed as, for viability, % viability compared to 100 % control cells, and as the ratio change compared to controls for LDH release, and each data point represents the mean ± SEM, R = 4; statistical significance (determined by ANOVA with Tukey posthoc) is shown by ^ = p < 0.05 for 24 h, and * = p < 0.05 for 48 h, compared to relevant controls. [file 12951_2015_146_MOESM2_ESM.pdf]

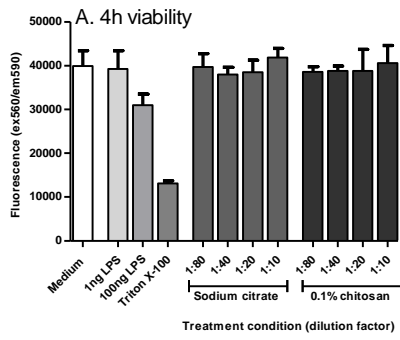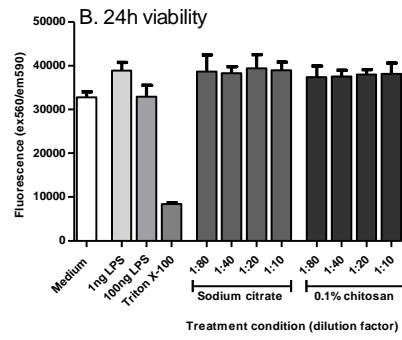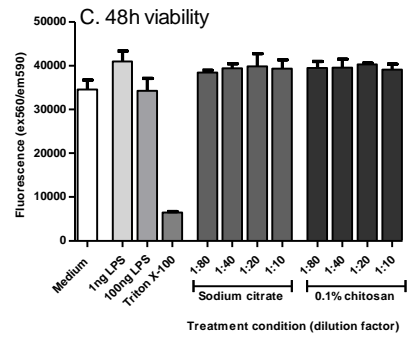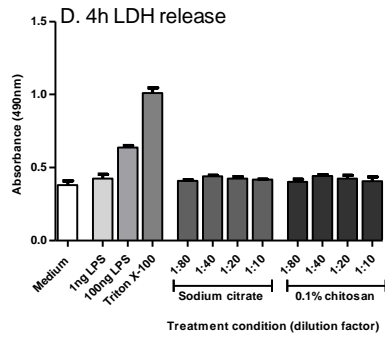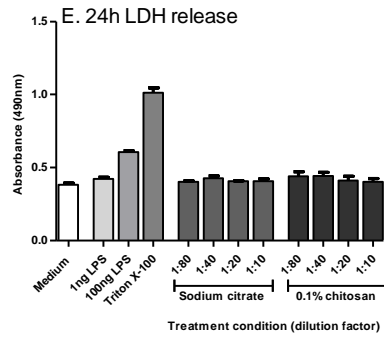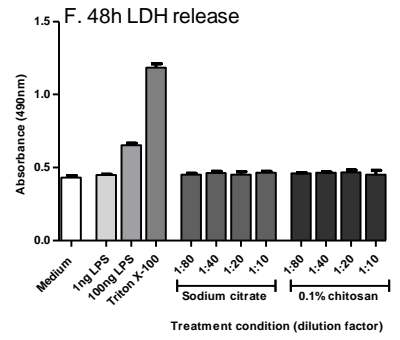

Supplement: Supplementary file 3 — 10.1186/s12951-015-0146-9 Cytotoxicity assessment of NP solvents in THP-1 cells. Viability (mitochondrial activity) (A-C) and cytotoxicity (LDH release) (D-F) in PMA-stimulated cells, after treatment with sodium citrate (2.2 mM stock) or chitosan (0.1 % stock) at dilutions relevant to AuNP exposures, for 4, 24 and 48 h; 1 ng/ml LPS was used for co-stimulation, Triton X-100 was used as positive control. Results are expressed as fluorescence intensity for viability assays, and as absorbance for LDH release, R = 3, each data point represents the mean ± SEM. [file 12951_2015_146_MOESM3_ESM.pdf]

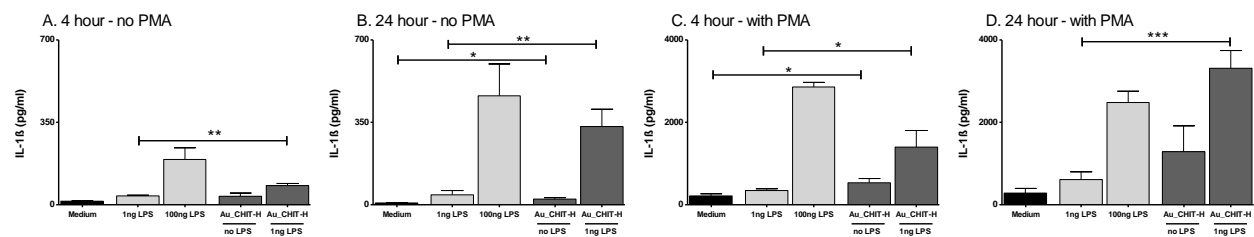

Supplement: Supplementary file 4 — 10.1186/s12951-015-0146-9 AuNPs induced pro-inflammatory response of THP-1 cells in different cell activation states. IL-1β release from THP-1 cells in response to treatment with Au_CHIT-H (administered dose of 2.5 µg/ml) for 4 (A and C) or 24 h (B and D), in the presence and absence of 1 ng/ml LPS co-stimulation, and without (A-B) or with (C-D) PMA-priming. Results are expressed as IL-1β release (pg/ml), and each data point represents the mean ± SEM, R = 3. Statistical significance is shown by * = p < 0.05, ** = p < 0.01 and *** = p < 0.005, compared to relevant controls (medium only or 1 ng/ml LPS); 100 ng/ml LPS was used as positive control. [file 12951_2015_146_MOESM4_ESM.pdf]

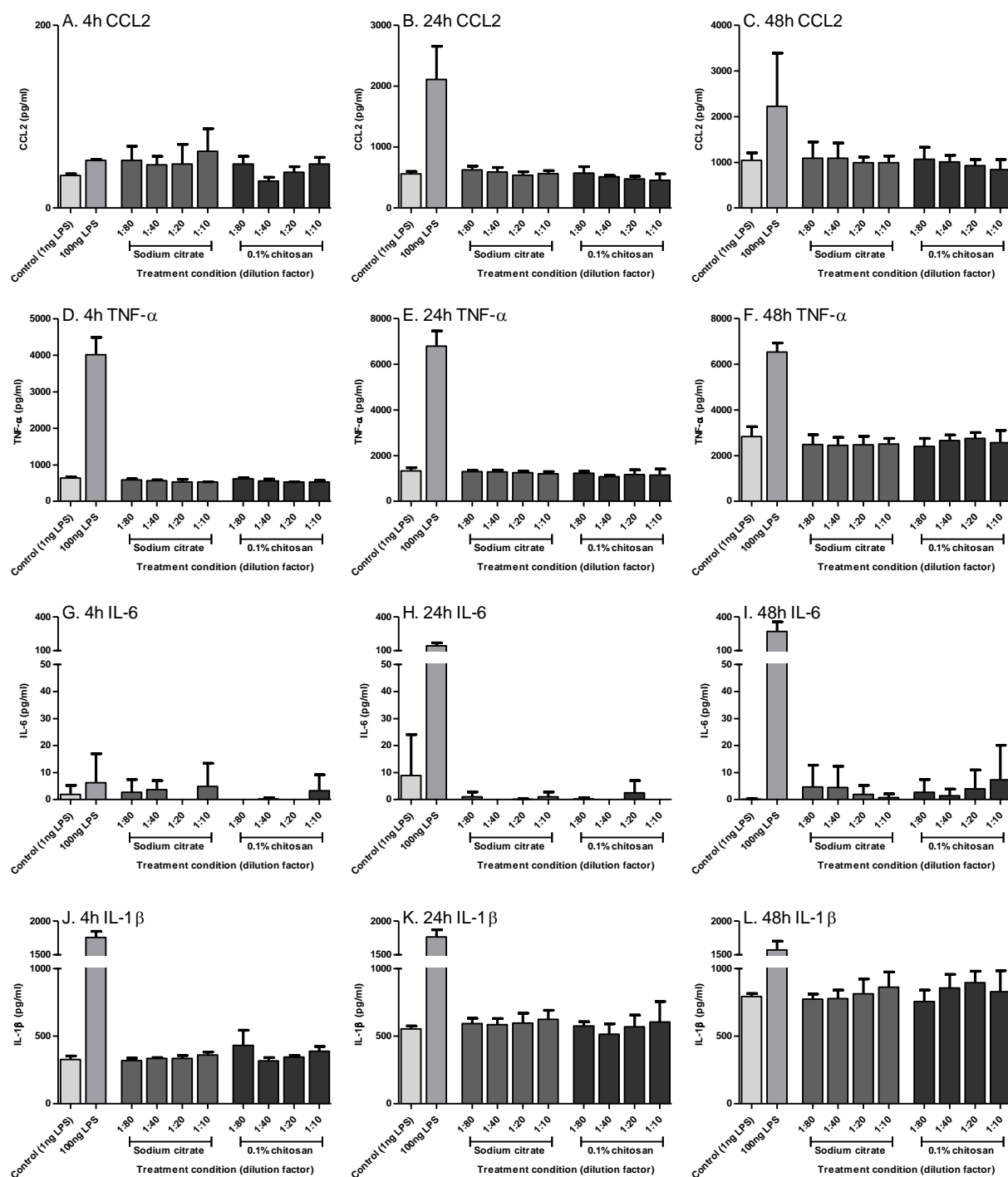

Supplement: Supplementary file 5 — 10.1186/s12951-015-0146-9 Immune response of THP-1 cells to NP solvents. Secretion of CCL2 (A-C), TNF-α (D-F), IL-6 (G-I) and IL-1ß (J-L) from PMA-stimulated cells, in response to sodium citrate (2.2 mM stock) or chitosan (0.1 % stock) at dilutions relevant to AuNP exposures; 1 ng/ml LPS was used for co-stimulation; with 1 and 100 ng/ml LPS for controls; results are expressed as cytokine release in pg/ml, R = 3, and each data point represents the mean ± SEM. [file 12951_2015_146_MOESM5_ESM.pdf]

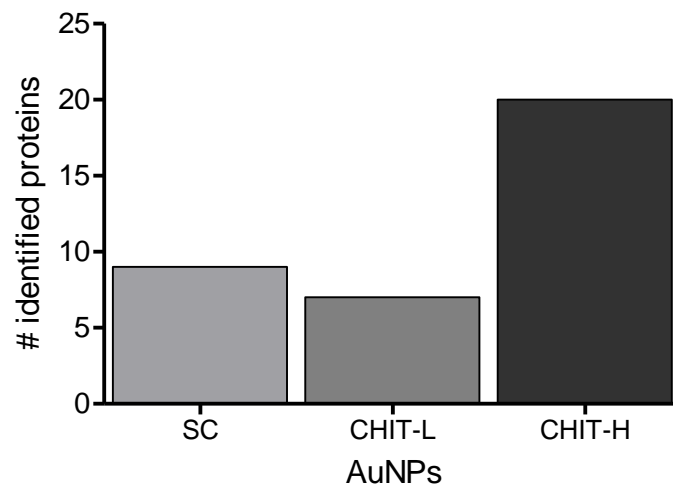

Supplement: Supplementary file 9 — 10.1186/s12951-015-0146-9 General pattern of AuNP-protein interactions – number of unique proteins. The number of unique proteins identified, via LTQ-Orbitrap mass spectrometry, in AuNP-FCS complexes were identified using http://www.uniprot.orf (taxonomy: mammalia); evaluated when incubated in 10, 55, and 100 % FCS; data presented is of unique proteins identified in every biological replicate and all 3 serum conditions. [file 12951_2015_146_MOESM9_ESM.pdf]
