# Supplementary material for: Chitosan functionalisation of gold nanoparticles encourages particle uptake and induces cytotoxicity and pro-inflammatory conditions in phagocytic cells, as well as enhancing particle interactions with serum components
Source: J Nanobiotechnology. 2015 Nov 18;13:84. doi: 10.1186/s12951-015-0146-9 (PMC4652435; doi:10.1186/s12951-015-0146-9)
Supplement: Supplementary file 7 — 10.1186/s12951-015-0146-9 Proteins bound to AuNP when using 55 % FCS. The identity of unique proteins identified, via LTQ-Orbitrap mass spectrometry, in AuNP-FCS complexes were identified using http://www.uniprot.org (taxonomy: mammalia); evaluated when incubated in 55 % FCS; data presented is of unique proteins identified in all 3 biological replicates; this analysis was performed with 3 technical replicates of each biological replicate; for confidence of identification the proteins presented in these graphs are only proteins found in every biological replicate. [file 12951_2015_146_MOESM7_ESM.pdf]

Au_SC	Au_CHIT-L	Au_CHIT-H
Serum albumin	Serum albumin	Serum albumin
Serotransferrin	Serotransferrin	Serotransferrin
Alpha-1-acid glycoprotein	Alpha-1-acid glycoprotein	Alpha-1-acid glycoprotein
Alpha-1-antiproteinase	Alpha-1-antiproteinase	Alpha-1-antiproteinase
Alpha-1B-glycoprotein	Alpha-2-HS-glycoprotein	Alpha-1B-glycoprotein
Alpha-2-HS-glycoprotein	Apolipoprotein A-I	Alpha-2-HS-glycoprotein
Hemoglobin fetal subunit beta	Hemoglobin fetal subunit beta	Hemoglobin fetal subunit beta
Vitamin D-binding protein	Vitamin D-binding protein	Vitamin D-binding protein
Apolipoprotein A-I		Apolipoprotein A-I
Apolipoprotein A-II		Apolipoprotein A-II
Transthyretin		Transthyretin
Serpin A3-1		Serpin A3-1
		Complement C3
Complement C4 (Fragments)		Complement C4 (Fragments)
		Complement factor B
		Complement factor H
		Apolipoprotein C-II
		Apolipoprotein C-III
		Apolipoprotein E
		Hemoglobin subunit alpha
		Hemoglobin subunit beta
		Histidine-rich glycoprotein (Fragments)
		Inter-alpha-trypsin inhibitor heavy chain H1
		Inter-alpha-trypsin inhibitor heavy chain H3
		Inter-alpha-trypsin inhibitor heavy chain H4
		Fetuin-B
		Acidic mammalian chitinase
		Adiponectin
		Antithrombin-III
		Clusterin
		Coagulation factor X
		Fibrinogen alpha chain
		Fibronectin
		Gelsolin
		Neuroendocrine secretory protein 55
		Pigment epithelium-derived factor
		Plasminogen
		Protein AMBP
		Prothrombin
		Thrombospondin-4
		Tubulin alpha-4A chain
