# Supplementary material for: Chitosan functionalisation of gold nanoparticles encourages particle uptake and induces cytotoxicity and pro-inflammatory conditions in phagocytic cells, as well as enhancing particle interactions with serum components
Source: J Nanobiotechnology. 2015 Nov 18;13:84. doi: 10.1186/s12951-015-0146-9 (PMC4652435; doi:10.1186/s12951-015-0146-9)
Supplement: Supplementary file 8 — 10.1186/s12951-015-0146-9 Proteins bound to AuNP when using 100 % FCS. The identity of unique proteins identified, via LTQ-Orbitrap mass spectrometry, in AuNP-FCS complexes were identified using http://www.uniprot.org (taxonomy: mammalia); evaluated when incubated in 100 % FCS; data presented is of unique proteins identified in all 3 biological replicates; this analysis was performed with 3 technical replicates of each biological replicate; for confidence of identification the proteins presented in these graphs are only proteins found in every biological replicate. [file 12951_2015_146_MOESM8_ESM.pdf]

Au_SC	Au_CHIT-L	Au_CHIT-H
Serum albumin	Serum albumin	Serum albumin
Serotransferrin	Serotransferrin	Serotransferrin
Alpha-1-acid glycoprotein	Alpha-1-acid glycoprotein	Alpha-1-acid glycoprotein
Alpha-1-antiproteinase	Alpha-1-antiproteinase	Alpha-1-antiproteinase
Alpha-2-HS-glycoprotein	Alpha-2-HS-glycoprotein	Alpha-1B-glycoprotein
Vitamin D-binding protein	Vitamin D-binding protein	Vitamin D-binding protein
Fetuin-B	Fetuin-B	Fetuin-B
Hemoglobin fetal subunit beta	Hemoglobin fetal subunit beta	Hemoglobin fetal subunit beta
		Hemoglobin subunit alpha
Apolipoprotein A-I	Apolipoprotein A-I	Apolipoprotein A-I
Apolipoprotein A-II		Apolipoprotein A-II
		Apolipoprotein C-II
		Apolipoprotein C-III
		Apolipoprotein E
	Complement C3	Complement C3
	Complement C4 (Fragments)	Complement C4 (Fragments)
		Complement factor B
	Pigment epithelium-derived factor	Pigment epithelium-derived factor
		Alpha-2-HS-glycoprotein
		Alpha-fetoprotein
		Aggrecan core protein
		Antithrombin-III
		Clusterin
		Fibronectin
		Inter-alpha-trypsin inhibitor heavy chain H4
		Serpin A3-1
		Transthyretin
