# Supplementary material for: Chitosan functionalisation of gold nanoparticles encourages particle uptake and induces cytotoxicity and pro-inflammatory conditions in phagocytic cells, as well as enhancing particle interactions with serum components
Source: J Nanobiotechnology. 2015 Nov 18;13:84. doi: 10.1186/s12951-015-0146-9 (PMC4652435; doi:10.1186/s12951-015-0146-9)
Supplement: Supplementary file 10 — 10.1186/s12951-015-0146-9 General pattern of AuNP-protein interactions – identity of unique proteins. The identity of unique proteins identified, via LTQ-Orbitrap mass spectrometry, in AuNP-FCS complexes were identified using http://www.uniprot.org (taxonomy: mammalia); evaluated when incubated in 10, 55, and 100 % FCS; data presented is of unique proteins identified in every biological replicate and all 3 serum conditions. [file 12951_2015_146_MOESM10_ESM.pdf]

Au_SC	Au_CHIT-L	Au_CHIT-H
Serum albumin	Serum albumin	Serum albumin
Serotransferrin	Serotransferrin	Serotransferrin
Alpha-1-acid glycoprotein	Alpha-1-acid glycoprotein	Alpha-1-acid glycoprotein
Alpha-1-antiproteinase	Alpha-1-antiproteinase	Alpha-1-antiproteinase
Hemoglobin fetal subunit beta	Hemoglobin fetal subunit beta	Hemoglobin fetal subunit beta
Vitamin D-binding protein	Vitamin D-binding protein	Vitamin D-binding protein
Alpha-1-acid glycoprotein	Alpha-1-acid glycoprotein	
Apolipoprotein A-I		Apolipoprotein A-I
Apolipoprotein A-II		Apolipoprotein A-II
		Complement C3
		Complement factor B
		Complement C4 (Fragments)
		Pigment epithelium-derived factor
		Hemoglobin subunit alpha
		Apolipoprotein E
		Antithrombin-III
		Clusterin
		Fibronectin
		Inter-alpha-trypsin inhibitor heavy chain H4
		Fetuin-B
